# On-Substrate Joule Effect Heating by Printed Micro-Heater for the Preparation of ZnO Semiconductor Thin Film

**DOI:** 10.3390/mi11050490

**Published:** 2020-05-10

**Authors:** Van-Thai Tran, Yuefan Wei, Hejun Du

**Affiliations:** 1 School of Mechanical and Aerospace Engineering, Nanyang Technological University, 50 Nanyang Avenue, Singapore 639798, Singapore; vanthai.tran@ntu.edu.sg; 2 Advanced Remanufacturing and Technology Centre, 3 Cleantech Loop, Singapore 637143, Singapore; wei_yuefan@artc.a-star.edu.sg

**Keywords:** inkjet printing, zinc oxide, heat treatment, micro-heater, semiconductor

## Abstract

Fabrication of printed electronic devices along with other parts such as supporting structures is a major problem in modern additive fabrication. Solution-based inkjet printing of metal oxide semiconductor usually requires a heat treatment step to facilitate the formation of target material. The employment of external furnace introduces additional complexity in the fabrication scheme, which is supposed to be simplified by the additive manufacturing process. This work presents the fabrication and utilization of micro-heater on the same thermal resistive substrate with the printed precursor pattern to facilitate the formation of zinc oxide (ZnO) semiconductor. The ultraviolet (UV) photodetector fabricated by the proposed scheme was successfully demonstrated. The performance characterization of the printed devices shows that increasing input heating power can effectively improve the electrical properties owing to a better formation of ZnO. The proposed approach using the on-substrate heating element could be useful for the additive manufacturing of functional material by eliminating the necessity of external heating equipment, and it allows in-situ annealing for the printed semiconductor. Hence, the integration of the printed electronic device with printing processes of other materials could be made possible.

## 1. Introduction

Exploration of inkjet printing for the fabrication of electronic devices has recently become a trendy research topic due to the remarkable advantages of the digital-additive fabrication such as saving material and time, high resolution, and compatibility with different materials [1,2]. These advantages of inkjet printing have been widely employed for the fabrication of semiconductor devices [3,4]. Thanks to its outstanding features and abundance, zinc oxide (ZnO) has attracted considerable attention and effort in the additive fabrication of electronics devices such as solar cells [5,6], photodetectors [3,7], and transistors [8,9]. Additive manufacturing of inorganic material from a precursor compound usually requires a heat treatment step in order to convert precursors to the required material [10,11]. However, the annealing process using an external furnace might restrain the progress of 3D printed integrative devices because of the added complexity of the fabrication system.

Low temperature processing of metal oxide has been intensively studied via different approaches, such as ultraviolet (UV) annealing [12,13] and laser sintering [14], in order to facilitate the formation of metal oxide by heating effect of high energy light beam, which requires high complexity setup for the processing system. Furthermore, direct use of nanoparticle ink was considered instead of precursor ink [15]. However, added surfactant to keep nanoparticle ink stable might be an issue for electronic application because it might change the properties of printed material. Alternatively, using local Joule heating to form metal oxide by thermal decomposition of precursor compound is an interesting approach to prepare small-size metal oxide pattern [16].

Joule heating is the phenomenon in which heat is generated from a conductive material when there is an electrical current run through the conductor. The power consumed is proportional to the square of the electrical current and the resistance of the conductor [17]. A micro-heater working on the Joule heating principle possesses advantages such as local heat and optimized energy consumption. Therefore, the resistive heater has been employed in application that requires localized heating and temperature control at small scale, such as activation of gas sensing device [18,19], moisture monitoring [20], and local growth of semiconductor nanostructures [21]. The traditional approach of preparing conductive patterns is using photolithography to deposit and remove certain parts of the conductive film and forming the required shape of the film. As this method has its own drawbacks, such as the high complexity and time-consuming, additive manufacturing could be a promising candidate for fabrication of micro-heaters [22].

In this work, a facile and versatile fabrication process for additive fabrication of semiconductor using inkjet printing and on-substrate heating was proposed. The printed conductive material is further employed for another role, which is the heating element for later processes. Zinc precursor ink is then printed on the same substrate. Eventually, electrical power was applied to the micro-heater to generate heat, which facilitates the decomposition of the zinc salt and the formation of ZnO. Therefore, the necessity for an external bulky furnace is eliminated. The generation of ZnO was examined by the elemental survey of zinc (Zn) and oxygen (O) component in energy-dispersive X-ray spectroscopy (EDS) and the aid of thermogravimetric analysis (TGA). In order to demonstrate the obtained semiconductor film, a UV photodetector application was prepared and characterized. The influence of electrical power during Joule heating to photodetector performance is also evaluated.

## 2. Materials and Methods

A commercialized Dimatix 2831 inkjet printer (Fujifilm Dimatix, Inc, Santa Clara, CA, USA) was employed in all printing steps described in this work using 10 pL cartridge with 16 nozzles. The silicon/silicon dioxide (Si/SiO_2_) substrate (Bonda Technology Pte Ltd, Singapore) was cleaned before the printing of silver ink to construct the electrodes and heater. The substrate was cleaned in acetone and rinsed with isopropanol, then it was dried out by a manual air blower.

**Figure A1:**
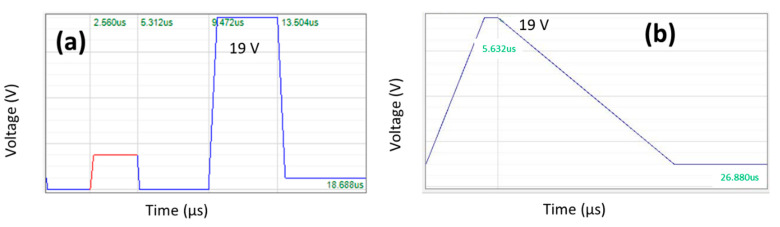
Waveform of signal applying to cartridge nozzles to create jetting of ink: (**a**) silver ink and (**b**) zinc salt ink.

A commercial silver nanoparticle ink (silver dispersion 736465, Sigma-Aldrich, St. Louis, MO, USA) was employed for the printing of the silver patterns. Detail of the printing step has been discussed in our previous report [23]. The waveform applied to piezoelectric nozzle to jet ink is shown in Figure A1a (Appendix A). Peak firing voltage was set at 19 V. Drop-spacing was set at 40 μm to ensure the continuity of the printed silver line. Single layer was selected for printing of electrodes and four-layers was selected for printing of micro-heater. Cartridge temperature was set at 35 °C, however, due to the printer platen temperature was set at 60 °C and the close distance of the cartridge and the substrate during printing, the cartridge temperature may rise to about 40 °C during printing. The printed pattern was left on the platen of the printer for 10 min for solvent vaporization.

Printed single line of conductive silver features 100 µm in width, and the thickness is about 200 nm as reported in our previous works [23]. The as-printed single line resistance was measured as 15.5 Ω. Due to the Joule heating process using 4-W power, the resistance was reduced to 5.0 Ω as the effect of sintering.

**Figure 1:**
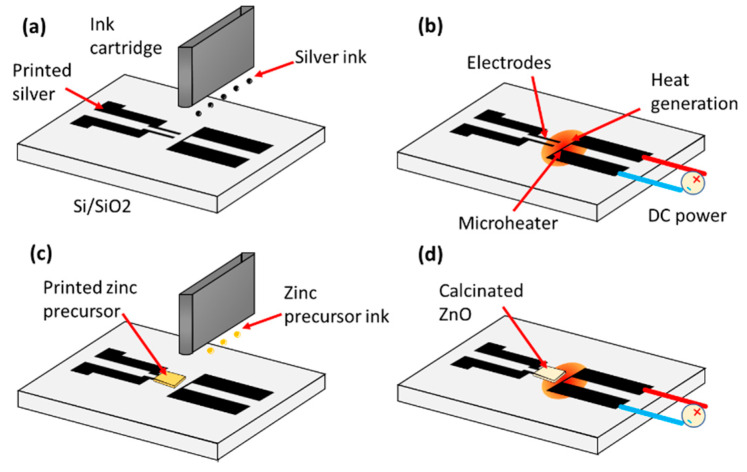
Fabrication step of the sensor using including printed micro-heater. (**a**) Printing of conductive pattern for the metal contacts and heater. (**b**) Applying direct current (DC) power to the micro-heater to generate heat and sinter silver. (**c**) Deposition of zinc salt ink over the printed electrodes. (**d**) Applying DC power again to the micro-heater to calcinate ZnO.

Figure 1 depicts the fabrication of the device using Joule heating. Printed metal patterns, serving as electrical contacts for the sensor as well as the micro-heater, was printed on the first step of the fabrication process (Figure 1a). The micro-heater composes of two contacting pad and a single line of conductive silver serving as a heating resistor. Then, direct current was applied to the micro-heater, which consumes 4-W power and converts it to heat energy for 5 min (Figure 1b). The generated heat promotes the sintering of silver electrodes and improves the bonding to substrate. The power applied to the micro-heater was manually adjusted by turning the voltage of the direct current (DC) power source when monitoring the current. During the Joule heat treatment, the resistance of the micro-heater changed due to sintering effect which require a careful adjustment of voltage. For example, a 5 W of power was obtained by turning the voltage to 5.0 V while the current reached 1.0 A.

A 50 mM zinc precursor solution was formulated by dissolving of zinc acetate dihydrate (Zn(CH_3_COO)_2_·2H_2_O) to ethanol. Another cartridge was used to print the zinc precursor after the replacement of the silver ink cartridge by zinc precursor ink cartridge. A rectangular pattern of the zinc salt solution is printed over the metal contacts, which is nearby the micro-heater (Figure 1c). The beneath substrate was kept heated at a temperature of 60 °C during printing to facilitate the evaporation of the solvent and reduce the spreading of solution over the surface.

Ten layers of zinc precursor was printed with a designed pattern of 60 pixels by 50 pixels. Zinc precursor ink printing parameters were optimized to ensure the stability during printing. The waveform was given in Figure A1b (Appendix A) with peak voltage at 19 V. The cartridge was kept at room temperature. Drop spacing, defining by the distance of two nearby droplets, was set at 10 μm, so that it is necessary to calibrate the cartridge holder angle during printing different materials.

After the printing of the precursor, electrical current was applied to the resistive heater again for 5 min (Figure 1d). Two sets of samples which have 4-W and 5-W applied power were studied along with the samples without the treatment. A thermal camera (NEC F30W, AVIO, Turin, Italy) was employed to measure the temperature of the device during the Joule heating process.

Thermogravimetric analysis (TGA) using the equipment TGA Q500 (TA Instruments, New Castle, DE, USA) was utilized for studying the formation of zinc oxide by thermal process. The heating rate we used for TGA is 10 °C/min. Film morphology and elemental study were characterized by field emission–scanning electron microscope (FE–SEM, JOEL 7600F, JEOL Ltd., Tokyo, Japan) and energy dispersive X-ray spectroscopy (EDS) (Oxford Instruments, Abingdon, UK). In order to characterize the performance of the Joule heating processed sensor, an ultraviolet light-emitting diode which emits 365 nm wavelength was used to illuminate the sensor and measure the photocurrent under dark or lighting condition. The photocurrent was recorded using a source metering unit (SMU B2902A, Agilent, Santa Clara, CA, USA).

## 3. Results

### 3.1. Sintering of Printed Silver

**Figure 2:**
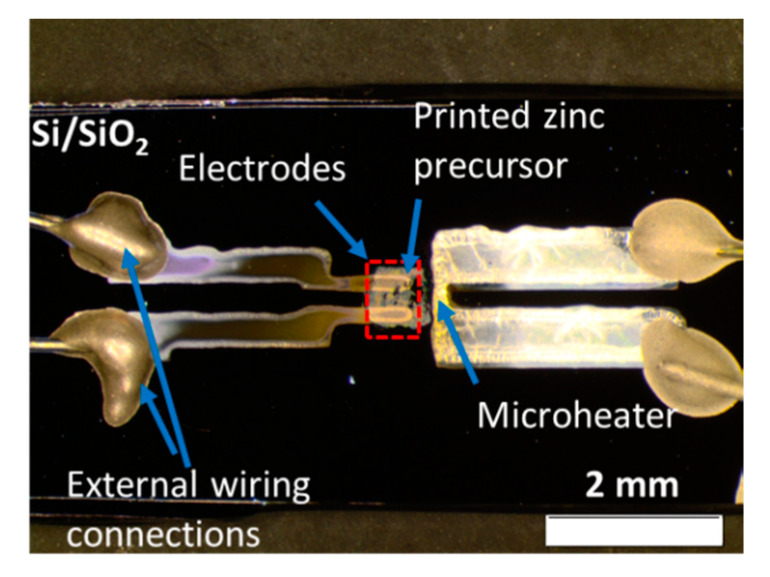
Optical image of the printed Joule heating device showing the three main components of the device, such as electrodes section, printed zinc salt film, and micro-heater. The picture was taken after the heat treatment of the ZnO pattern.

**Figure A2:**
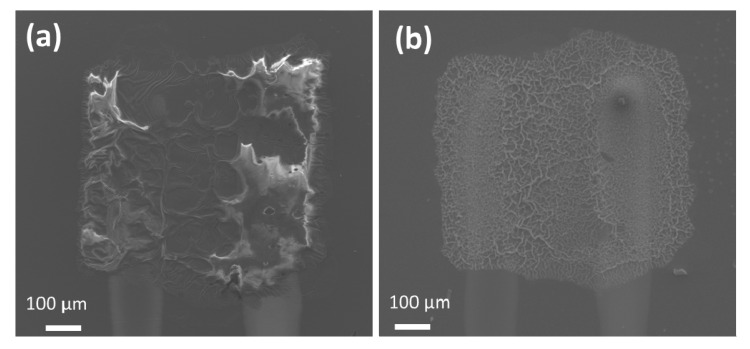
Low-magnification FE–SEM images of two samples before (**a**) and after heat treatment (**b**).

The structure of the device is depicted in Figure 2, which shows three main components of the devices deposited on Si/SiO_2_ substrate. While the common electrodes and the micro-heater were printed on the first layer, the zinc precursor was printed at last. The zinc precursor appears as a white rectangle pattern over the silver electrodes. Thus, the photodetector utilizes the metal-semiconductor contacts with the two-terminals structure. Two low-magnification FE–SEM images of samples before and after heat treatment was presented in Figure A2 (Appendix A) to provide a better understanding of the effect of heat treatment to the film.

**Figure 3:**
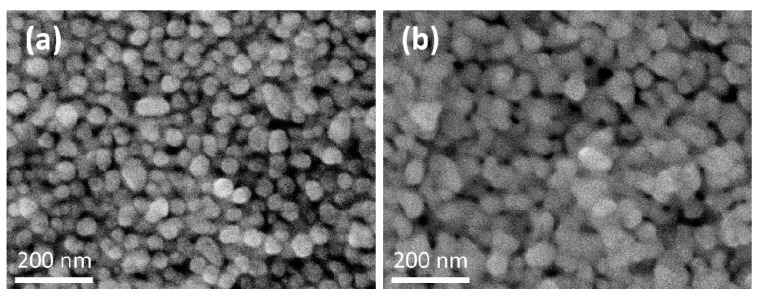
Field emission–scanning electron microscope (FE–SEM) images show the sintering of printed silver nanoparticles ink by the resistive heating. (**a**) The printed silver nanoparticles without annealing. (**b**) The silver nanoparticles at electrode section after annealing.

The effect of Joule heating on the nanostructure of printed silver could be observed in Figure 3, which shows the sintering of silver nanoparticles. Printed silver film composes of separated particles after the evaporation of the solvent. The sintering effect is significantly depending on the distance from the heater. At the electrode section (Figure 3b), which is distinct from the radiation source, the particle size of approximately 80 to 100 nm could be observed, which is an increase from the size of about 50 nm of the unsintered particles (Figure 3a).

**Figure 4:**
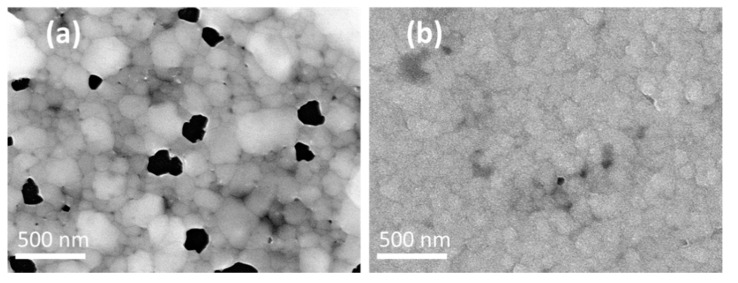
FE–SEM images show the sintering of micro-heater with different numbers of printed silver layers: (**a**) single-layer printing and (**b**) four-layer printing.

Heating radiation from resistive effect is the main source that induces the sintering of the printed silver pattern. In Figure 4a, the heater witnesses a notable change of film morphology, such as particle agglomeration up to the size of 200 nm. As the previous discussion has pointed out, the remote pattern exhibits minor sintering effect, while at the center of heat source, major agglomeration could be observed. Later analysis of temperature distribution shows that this is indeed a result of gradually reduce of temperature in the substrate. Although the sintering of silver could improve the electrical properties of the conductive pattern, a severe agglomeration of silver nanoparticles could lead to the interruption of the conductive track and open the circuit. In order to reach a sufficient temperature for the later heat treatment of the sensing material, multi-layers printing of silver was carried on. The heat treatment was conducted after all the layers were printed to reduce dislocation of printed layers due to handling during heat treatment. Figure 4b shows a notable improvement in film morphology when four-layer printing was employed, which does not exhibit severe agglomeration and any gap in film after heat treatment.

### 3.2. Temperature Survey of Joule Heating

**Figure 5:**
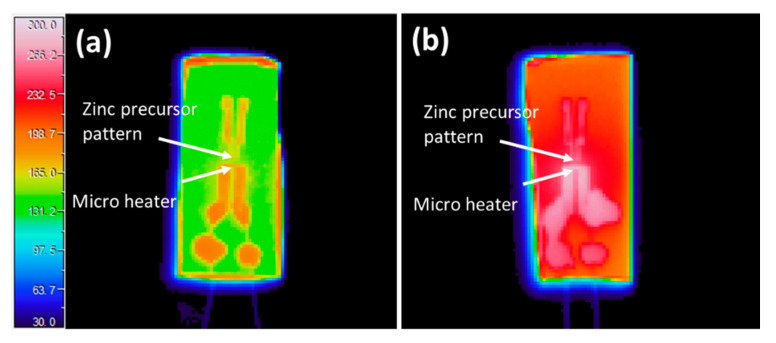
Temperature survey of the device with different heating power by the thermal camera. (**a**) 4-W electrical power. (**b**) 5-W electrical power. The unit of temperature scale bar is degree Celsius.

The temperature of the device during the Joule heating was investigated using the thermal camera and the results are presented in Figure 5. Thermal photo was taken from the back of the device to avoid the complexity of different emissivity of materials. The temperature was calibrated using the software provided by the thermal camera’s manufacturer (NS9205 Viewer, Avio) with applied emissivity of SiO_2_ of 0.9 [24].

After calibration using emissivity of 0.9 of SiO_2_, an average temperature of 184 °C could be measured at the resistive heater, while that at the zinc precursor film was 171 °C when input electrical power was set at 4 W. There was a significant increase of temperature when raising the input power. When the input power was set at 5 W, the recorded average temperature is 267 °C at the heater and 239 °C at the precursor pattern. The temperature of the resistive heater depends on the input power, and their correlation might possibly be expressed by the relation [25]:

*P* = *a*(*T* − *T*_0_) + *b*(*T* − *T*_0_)^2^ + *c*(*T*^4^ − *T*_0_^4^), (1)

where *P*, *T,* and *T*_0_ are input power, heater temperature and ambient temperature, respectively, and *a*, *b,* and *c* are fitting parameters.

### 3.3. Generation of ZnO by Joule Heating

**Figure 6:**
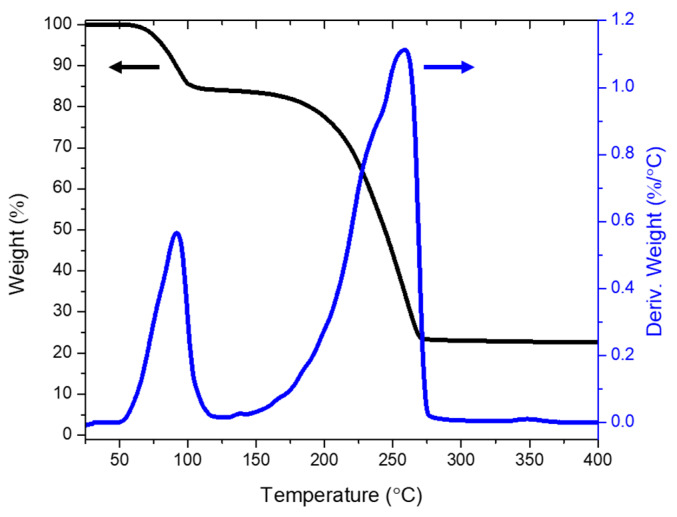
Thermogravimetric analysis (TGA) survey of zinc precursor in the air to study the formation of ZnO.

Thermogravimetric analysis (TGA) was utilized to investigate the calcination of the precursor and formation of ZnO by high-temperature treatment, which could be cataloged into two stages: the vaporization stage and the decomposition of zinc precursor stage [26]. Figure 6 shows the result of thermal analysis of zinc acetate dihydrate in ambient air. When temperature raising from 60 to 100 °C, water vaporization occurs, which could be correlated to the sharp decline of the salt weight of 15% in TGA result. There is a slight decay from 150 to 200 °C, which denotes the starting of decomposition process. From 200 °C, there is a considerable reduction of weight as the reaction is promoted by temperature. The weight is stable at approximately 23% after 370 °C, indicating that the thermo-decomposition has completed and most of zinc salt has been transformed to ZnO. Although this result recommends that temperature of above 370 °C is necessary to thoroughly decompose the zinc precursor, it also suggests that lower temperature still could partially form ZnO. Starting with zinc acetate dihydrate, the reaction is finished with most of the products of the process are volatile, such as water, acetone ((CH_3_)_2_CO), acetic acid (CH_2_COOH), and carbon dioxide (CO_2_) [27].

Elemental and morphological studies of the heat-treated film could provide more evidence for the mechanism of the generation of ZnO. Because the decomposition reaction of zinc acetate dihydrate shows the loss of oxygen element via product vaporization, an investigation of Zn:O atomic ratio would be meaningful for tracking the formation of ZnO. Using EDS analysis, which is shown in Table A1 (Appendix A), it was found that the as-printed film exhibits an average Zn:O atomic ratio of 0.378. In the case of using 4-W Joule heating, the atomic ratio of 0.588 could be observed. This Zn:O atomic ration further increases to 0.605 when 5-W heating power was applied. The rise of Zn:O atomic ratio is an important evidence of thermal decomposition reaction and the formation of ZnO by Joule heating. It could be seen that the Zn:O atomic ratio is not significantly different between 4- and 5-W heating power. Furthermore, there is a noticeable variation in this atomic ratio investigation through the pattern, which could be contributed to the grading of temperature through the surface.

**Figure 7:**
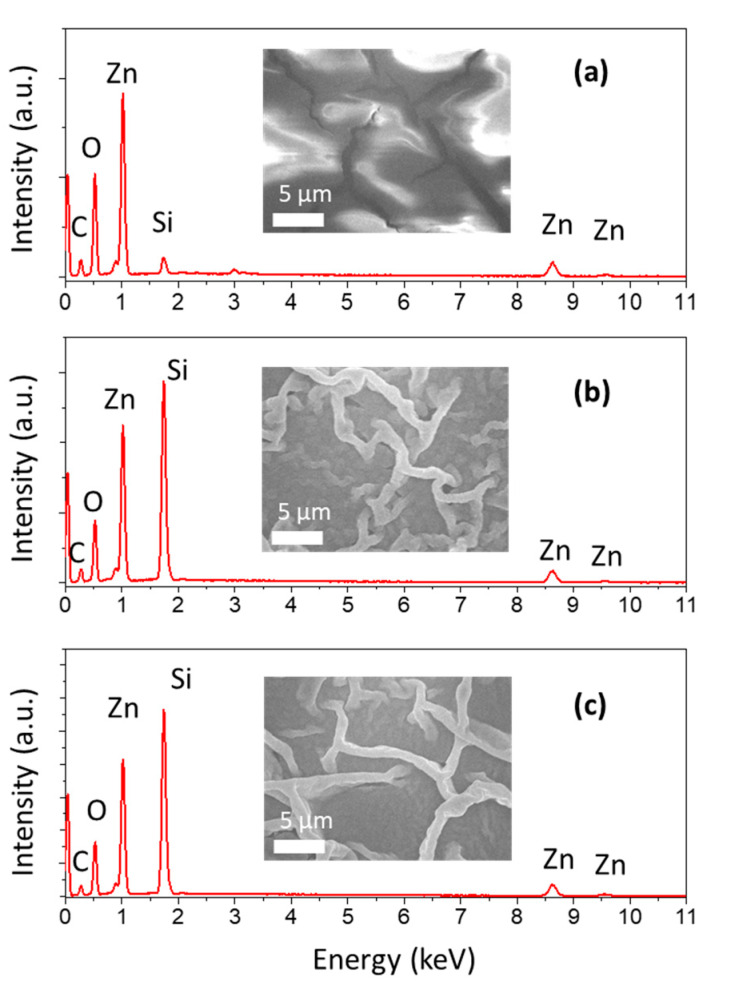
Energy-dispersive X-ray spectroscopy (EDS) analysis of zinc precursor film with different treatment conditions: (**a**) 0 W, (**b**) 4 W, (**c**) 5 W. Insets are FE–SEM images of film morphology according to each condition.

Furthermore, morphological study depicts a remarkable change in film structure after Joule heating process (Figure 7). Figure 7a demonstrates the zinc precursor film before any heat treatment was applied. The film appears to be full of fractures, which could be the result of the solvent evaporation and the condensation of salt. There are remarkable changes in film morphology after the Joule heating treatment, such as the vanishing of notable fractures and the appearance of wrinkles on the surface of the film (Figure 7b,c). However, there is no significant difference between these two treated films. These film structures are commonly observed in the sol-gel derived film as our previous report [3]. These wrinkles could be originated from the internal stress of film during rapid solvent withdrawal, caused by the difference in thermal expansion of the gelation and underlying layer [28]. In addition, the transition from viscous to viscoelastic of the zinc precursor ink also contributes to the formation of these wrinkles [29].

### 3.4. UV Light Sensing Performance of the Sensor

**Figure 8:**
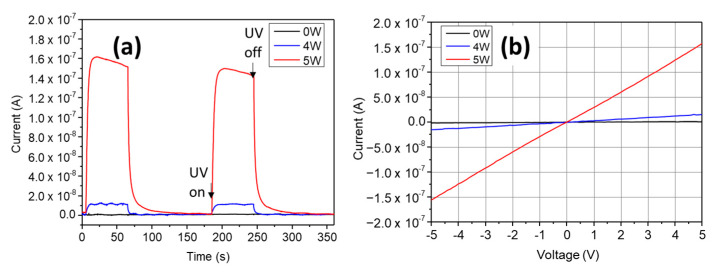
Photo-sensing properties of the fabricated device. (**a**) Sequential illumination of the sensor by UV light and obtained photocurrent. (**b**) *I–V* curve of the devices under UV illumination.

The sensing of UV light was demonstrated by the fabricated device and the result was presented in Figure 8. A bias voltage of 5 V was applied to the two terminals of device, which also serves as electrode by making metal contacts with the semiconductor film. The electrical current was recorded while the UV light was turned on/off (Figure 8a). It could be noted that the sample without applying heat treatment did not shows a notable response to UV light. On the other hand, sample with the treatment exhibits remarkable response to the short-wavelength light. When UV was turn on, the current raised significantly until it reached the equilibrium. On the other hand, when UV was turned off, the current quickly decay to the initial value. It is also worth noting that there is a remarkable difference of the samples with different heating conditions, such as the sample treated with 5-W power has photocurrent with a maximum value of 1.6 × 10^−7^ A, which is about ten times higher than that of the sample treated with 4-W power.

The responsivity could be calculated using following expression: [30]

(2) $$R=\frac{J_{ph}}{PS}$$

where *Jph* is the photocurrent, *P* is the light intensity, and *S* is the effective area, which could be determined by the area of the ZnO pattern between two electrodes, such as 0.2 mm × 0.6 mm. Further analysis shows the responsivity values at 5.42 mW/cm^2^ light intensity of the 5-W sample and the 4-W sample is 0.029 and 0.0027 A/W, respectively. Although the responsivity is lower than that has been reported in other inkjet-printed ZnO-based UV photodetector [31], it could be due to the obtained temperature is lower than the point where the reaction is totally finished. In Figure 8b, the I–V characteristics of prepared devices were presented, which show a linear current–voltage relationship for both of the devices with treatment. This behavior indeed shows the Ohmic contact nature of the silver–ZnO interface.

## 4. Discussion

The photosensitivity of the device is strongly influenced by the heat treatment condition. As the discussion in previous section has pointed out, temperature of higher than 150 °C is critical to facilitate the reaction to form ZnO from the zinc salt. It is worth noting that photosensitivity is a common characteristic of a semiconductor. ZnO is a wide bandgap semiconductor (*Eg* = 3.35 eV at room temperature [32]). Therefore, it only be sensitive with larger photon energy of the incident light, such as the UV light used in this work which has photon energy of 3.40 eV according to 365 nm central wavelength of the LED.

The sample without heat treatment could not form ZnO by the thermal decomposition process. Therefore, the prepared film failed to work as a photodetector. Meanwhile, the sample processed with 4-W power demonstrates a remarkable response to UV illumination. It is because the temperature generated by the Joule heating process has facilitated the formation of ZnO semiconductor. As a wide bandgap semiconductor, the interaction of ZnO crystalline with high energy photons excites the generation of the electron-hole pair which increases the carrier concentration in the lattice [33]. Therefore, film resistance reduces and causes a surge in current running through the device.

Furthermore, the sample processed with 5-W power possesses a better performance in terms of responsivity to UV light. This improvement indicates that the magnitude of input power for Joule heating of semiconductor film could significantly influence the film properties. The root of this enhancement could be originated from the fact that higher temperatures could promote more formation of ZnO as previous discussion, therefore the interaction with photon is improved and more electron-hole pairs could be generated when exposing to the UV illumination. In addition, higher temperatures could also improve the contact between ZnO nanoparticles, therefore reduce the band bending at the interfaces and promote the transportation of electron through those grain boundaries [30].

Current work employed silicon wafer due to its excellent heat resistance, because of the temperature during Joule heating could excess 250 °C, which most of polymer will not be able to withstand. One possible alternative solution is using high temperature polymer such as polyimide. However, due to the low thermal conductivity of polyimide comparing that of silicon wafer [34,35], the structure of device must be changed to reduce the distance from heater to the printed zinc salt in order to obtain a sufficient calcination.

## 5. Conclusions

In this work, on-substrate heating synthesis of ZnO thin film by the printed silver resistive heater is proposed. Both conductive patterns and the precursor pattern were printed on only one inkjet printer with an exchange of cartridge for each material. Electrical current running through the silver conductive pattern generates heat, which is utilized to facilitate the thermal decomposition of printed zinc precursor film to form ZnO. The magnitude of supplied power for heating process has significant influence on the film formation via the achieved temperature. Therefore, it is found that higher power could produce a better property of printed ZnO semiconductor film. Despite the humble performance of printed photodetector, this work demonstrates a promising approach to additively manufacture electronic devices, which reduces the number of equipment involved and the amount of energy consumed. Hence, integrated 3D printing might also be possible.

## Appendix A

**Table A1:** EDS Investigation of Zn:O atomic ration at random positions on printed pattern.

	Zn:O Atomic Ratio							
Location	1	2	3	4	5	6	Average	Standard Deviation
0 W	0.430	0.431	0.422	0.390	0.292	0.305	0.378	0.058
4 W	0.536	0.545	0.504	0.705	0.681	0.557	0.588	0.076
5 W	0.553	0.567	0.517	0.691	0.651	0.648	0.605	0.062
